# Supplementary figures and images for: Mating-Type Analysis in Diaporthe Isolates from Soybean in Central Europe
Source: J Fungi (Basel). 2025 Mar 25;11(4):251. doi: 10.3390/jof11040251 (PMC12028000; doi:10.3390/jof11040251)

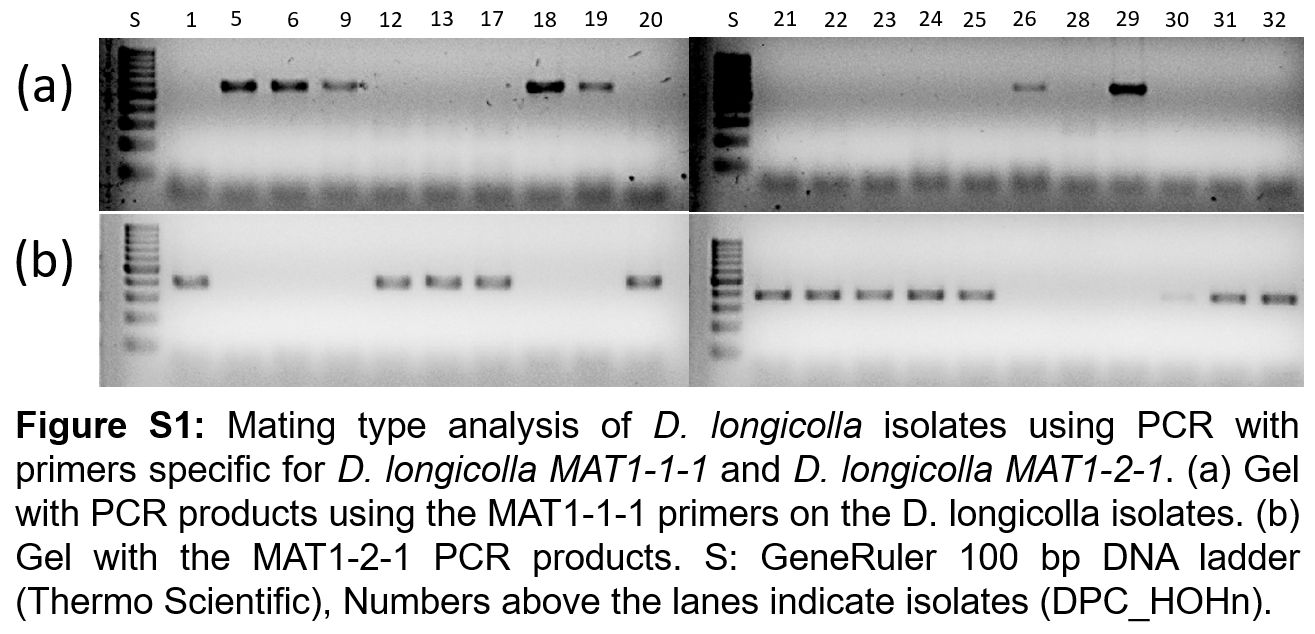

Supplement: Supplementary file 1 [file jof-11-00251-s001.zip › Figure S1.PNG]

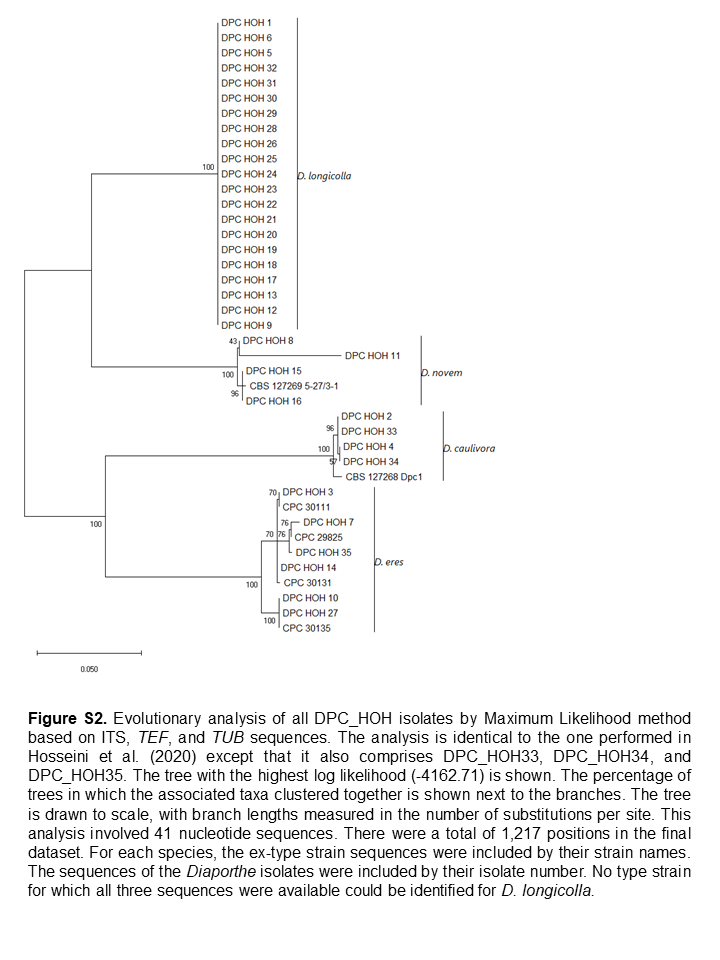

Supplement: Supplementary file 1 [file jof-11-00251-s001.zip › Figure S2.png]
